# DNA methylation and general psychopathology in childhood: an epigenome-wide meta-analysis from the PACE consortium

**DOI:** 10.1038/s41380-022-01871-6

**Published:** 2022-11-16

**Authors:** Jolien Rijlaarsdam, Marta Cosin-Tomas, Laura Schellhas, Sarina Abrishamcar, Anni Malmberg, Alexander Neumann, Janine F. Felix, Jordi Sunyer, Kristine B. Gutzkow, Regina Grazuleviciene, John Wright, Mariza Kampouri, Heather J. Zar, Dan J. Stein, Kati Heinonen, Katri Räikkönen, Jari Lahti, Anke Hüls, Doretta Caramaschi, Silvia Alemany, Charlotte A. M. Cecil

**Affiliations:** 1 Department of Child and Adolescent Psychiatry/ Psychology, Erasmus MC University Medical Center Rotterdam, Rotterdam, The Netherlands; 2 ISGlobal, Barcelona Institute for Global Health, Barcelona, Spain; 3 Universitat Pompeu Fabra, Barcelona, Spain; 4 Centro de investigación biomédica en red en epidemiología y salud pública (ciberesp), Madrid, Spain; 5 School of Psychological Science, MRC Integrative Epidemiology Unit, University of Bristol, Bristol, UK; 6 Institute for Sex Research, Sexual Medicine and Forensic Psychiatry, University Medical Center Hamburg, Eppendorf, Germany; 7 Department of Epidemiology, Rollins School of Public Health, Emory University, Atlanta, GA, USA; 8 Department of Psychology & Logopedics, University of Helsinki, Helsinki, Finland; 9 VIB Center for Molecular Neurology, Antwerp, Belgium; 10 The Generation R Study Group, Erasmus MC University Medical Center Rotterdam, Rotterdam, The Netherlands; 11 Department of Pediatrics, Erasmus MC University Medical Center Rotterdam, Rotterdam, The Netherlands; 12 Division of Climate and Environmental Health, Norwegian Institute of Public Health (NIPH), Oslo, Norway; 13 Department of Environmental Science, Vytautas Magnus University, 44248 Kaunas, Lithuania; 14 Bradford Institute for Health Research, Bradford Teaching Hospitals NHS Foundation Trust, Bradford, UK; 15 Department of Social Medicine, University of Crete, Crete, Greece; 16 Department of Paediatrics and Child Health, Red Cross War Memorial Children’s Hospital, University of Cape Town, Cape Town, South Africa; 17 South African Medical Research Council (SAMRC) Unit on Child and Adolescent Health, University of Cape Town, Cape Town, South Africa; 18 Department of Psychiatry and Mental Health, University of Cape Town, Cape Town, South Africa; 19 South African Medical Research Council (SAMRC) Unit on Risk and Resilience in Mental Disorders, Neuroscience Institute, University of Cape Town, Cape Town, South Africa; 20 Psychology/ Welfare Sciences, Faculty of Social Sciences, Tampere University, Tampere, Finland; 21 Gangarosa Department of Environmental Health, Rollins School of Public Health, Emory University, Atlanta, GA, USA; 22 Medical Research Council Integrative Epidemiology Unit, Population Health Science, Bristol Medical School, University of Bristol, Bristol, UK; 23 Department of Psychology, University of Exeter, Exeter, UK; 24 Psychiatric Genetics Unit, Group of Psychiatry, Mental Health and Addiction, Vall d’Hebron Research Institute (VHIR), Universitat Autònoma de Barcelona, Barcelona, Spain; 25 Biomedical Network Research Centre on Mental Health (CIBERSAM), Instituto de Salud Carlos III, Barcelona, Spain; 26 Department of Epidemiology, Erasmus MC University Medical Center Rotterdam, Rotterdam, The Netherlands; 27 Molecular Epidemiology, Department of Biomedical Data Sciences, Leiden University Medical Center, Leiden, The Netherlands

## Abstract

The general psychopathology factor (GPF) has been proposed as a way to capture variance shared between psychiatric symptoms. Despite a growing body of evidence showing both genetic and environmental influences on GPF, the biological mechanisms underlying these influences remain unclear. In the current study, we conducted epigenome-wide meta-analyses to identify both probe- and region-level associations of DNA methylation (DNAm) with school-age general psychopathology in six cohorts from the Pregnancy And Childhood Epigenetics (PACE) Consortium. DNAm was examined both at birth (cord blood; prospective analysis) and during school-age (peripheral whole blood; cross-sectional analysis) in total samples of *N* = 2178 and *N* = 2190, respectively. At school-age, we identified one probe (cg11945228) located in the Bromodomain-containing protein 2 gene (*BRD2*) that negatively associated with GPF (*p* = 8.58 × 10^–8^). We also identified a significant differentially methylated region (DMR) at school-age (*p* = 1.63 × 10^–8^), implicating the SHC Adaptor Protein 4 (*SHC4*) gene and the EP300-interacting inhibitor of differentiation 1 (*EID1*) gene that have been previously implicated in multiple types of psychiatric disorders in adulthood, including obsessive compulsive disorder, schizophrenia, and major depressive disorder. In contrast, no prospective associations were identified with DNAm at birth. Taken together, results of this study revealed some evidence of an association between DNAm at school-age and GPF. Future research with larger samples is needed to further assess DNAm variation associated with GPF.

## Introduction

Psychiatric disorders or symptoms co-occur more often than would be expected by chance alone [1, 2]. In light of the negative clinical and functional outcomes associated with psychiatric co-occurrence [3, 4], it is important to identify early indicators of risk and underlying biological mechanisms. There is accumulating evidence that, as early as in childhood, the shared variance between psychiatric disorders or symptoms may be usefully represented by a general psychopathology factor (GPF) [5–8]. This GPF in childhood has been found to show temporal stability [6] and to predict long-term functional and psychiatric outcomes in adolescence throughout adulthood [9, 10]. Although previous research has found evidence for both genetic and environmental influences on GPF [7, 11–16], the biological mechanisms underlying these influences remain unclear.

One of the ways by which genetic and environmental factors might contribute to disease susceptibility is through epigenetic mechanisms that regulate gene expression, such as DNA methylation (DNAm) [17]. Studies have shown that variation in DNAm is influenced by a dynamic interplay of genetic and environmental factors [18]. In turn, alterations in DNAm patterns across the genome in peripheral tissues including cord blood, and peripheral blood have been found to associate with a wide range of child and adult mental health outcomes, such as conduct problems, attention deficit hyperactivity disorder (ADHD) symptoms, major depressive disorder (MDD), and schizophrenia [19–22]. Despite a growing body of research implicating an involvement of DNAm in individual mental health outcomes, much less work has focused on the relationship between DNAm and general psychopathology [23]. To the best of our knowledge, only one study examined the association between genome-wide DNAm patterns and GPF in childhood. In this study, data were analyzed cross-sectionally in one cohort, focusing on wider biological networks (so called ‘modules’) of co-methylated DNAm probes across the genome [23]. As such, we still lack knowledge on how GPF relates to single DNAm probes and the extent to which associations vary across time (i.e., both cross-sectional and prospective associations). Large multi-cohort epigenome-wide studies, which allow for increased power and generalizability, are needed to improve our understanding of the biological mechanisms underlying shared variance across mental health problems.

We conducted epigenome-wide meta-analyses to investigate both probe-level and region-based associations of DNAm with school-age GPF in the Pregnancy And Childhood Epigenetics (PACE) Consortium. Because it is unclear at which time point differential DNAm may be most relevant to GPF, we examined DNAm both at birth (cord blood; prospective study; pre-symptom manifestation) and at school-age (peripheral whole blood; cross-sectional study) in pooled samples of *N* = 2178 and *N* = 2190 children, respectively.

## Methods

### Participants

The prospective analyses included four cohorts from PACE, using complete data on DNAm at birth, general psychopathology in childhood and covariates: the Avon Longitudinal Study of Parents and Children (ALSPAC), Drakenstein Child Health Study (DCHS), Generation R (GENR), and INfancia y Medio Ambiente (INMA). These cohorts have a combined sample size of 2178 (see Table 1). All prospective cohorts included participants of European ancestry, except for DCHS, which included participants of predominantly Black African ancestry or mixed ancestry. See Supplementary Methods for full cohort descriptions.

The cross-sectional analyses included four cohorts from the PACE consortium, using complete data on DNAm and general psychopathology in childhood, as well as covariates; ALSPAC, GENR, Glycyrrhizin in Licorice (GLAKU), and Human Early Life Exposome (HELIX; including six jointly analyzed sub cohorts). These cohorts have a combined sample size of 2190 (see Table 1). All cross-sectional cohorts included participants of European ancestry, except for HELIX, which included participants of European ancestry and participants with a Pakistani background living in the United Kingdom, which were analyzed as separate cohorts in our meta-analysis.

### Measures

#### DNA methylation

DNAm was assessed with the Illumina Infinium HumanMethylation450 (ALSPAC, DCHS, GENR, HELIX, INMA) or the Infinium HumanMethylationEPIC (DCHS, GLAKU) BeadChip assays in cord blood and in peripheral whole blood at ages 7–12 years. The cohorts performed sample processing, quality control (QC) and normalization based on their preferred protocols as described in the Supplementary methods. We used normalized, untransformed beta values, ranging from 0 (fully unmethylated) to 1 (fully methylated). Methylation levels that fell outside of the lower quartile minus 3×interquartile or upper quartile plus 3×interquartile range were removed.

We excluded probes with a call rate <90%, control probes, and probes that mapped to X/Y chromosomes. Following Zhou et al. [24], we further excluded probes with poor base pairing quality (lower than 40 on 0–60 scale), probes with non-unique 30 bp 3’-subsequence (with cross-hybridizing problems), Infinium II probes with SNPs of global MAF over 1% affecting the extension base, and probes with a SNP in the extension base that causes a color channel switch from the official annotation. We also excluded a subset of probes (n = 69) that have shown to be unreliable in a recent comparison of the Illumina 450 K and EPIC BeadChips [25]. At the meta-analysis level, we excluded probes which were available in <50% of the cohorts and <50% of the participants. After QC, 404,017 probes remained at birth and 413,497 probes remained at school-age.

#### General psychopathology factor

Mental health symptoms were assessed when children were aged 6–12 years, depending on the cohort. Parentrated instruments were used, including (i) the Child Behavior Checklist 6–18 (CBCL/6–18) in DCHS, GENR, GLAKU, HELIX, and INMA, and (ii) the Development and Well-being Assessment (DAWBA) in ALSPAC. Instruments are described in more detailed in the Supplementary methods. Whereas a single general factor loaded on all CBCL or DAWBA problem subscales, two specific factors loaded on internalizing (CBCL: anxious/ depressed, withdrawn/depressed, somatic complaints; DAWBA: generalized anxiety disorder, major depressive disorder, social phobia, separation anxiety, specific phobia) versus externalizing (CBCL: rule-breaking behavior, aggressive behavior, attention problems; DAWBA: attention deficit hyperactivity disorder, oppositional defiant disorder, conduct disorder) subscales. For the CBCL, three subscales (social problems, thought problems, other problems) were indicators of the general factor but were not part of the specific internalizing or externalizing factors. Of note, GLAKU included only two of these three CBCL subscales as the ‘other problems’ subscale was not available.

The internalizing and externalizing factors were allowed to correlate with each other but not with the general factor. As such, the general factor represents the shared variance among all problem scales that is independent of the more specific internalizing and externalizing factors. Previous research reported negative correlations between the GPF and cognitive outcomes [10, 14, 26]. To support the criterion validity of the GPF, we estimated the correlation between the GPF and child cognition across the cohorts.

#### Covariates

We adjusted for the following potential confounders: child sex, gestational age at birth, child age at the assessment of outcome, maternal age, maternal educational level, prenatal maternal smoking status, cell-type proportions estimated using standard algorithms for DNAm at birth [27] or childhood [28], ancestry (depending on the specific cohort), and technical covariates (e.g., batch) (see Supplementary methods). To test the robustness of findings when using a different method to estimate cell-type proportions, we re-ran the cross-sectional EWAS analyses within the cohort with the largest sample size (HELIX, itself comprised of different participating cohorts) with newly estimated cell proportions using IDOL (Salas et al., 2018 [29]) instead of Houseman’s approach [28].

### Statistical analyses

#### General psychopathology factor

We used confirmatory factor analysis (CFA) to fit a general psychopathology model in the full samples with mental health data available (see Supplementary Information 1). Each cohort ran the CFA according to a predefined script, using the Lavaan statistical package [30] in R (https://www.r-project.org/). GPF scores were extracted, winsorized at +/- 3SD, and standardized.

#### Cohort-specific EWAS

Each cohort ran the EWAS according to a predefined analysis plan, using robust linear regression (rlm; *MASS* R-package) to account for potential heteroscedasticity and non-normality. Cohorts excluded all multiple births and chose one random sibling per non-twin sibling pair.

#### Meta-analysis

The cohort-specific results were meta-analyzed at Erasmus MC Rotterdam. A shadow meta-analysis was conducted independently at the Barcelona Institute for Global Health. We performed an inverse-variance weighted fixed effects approach using R and METAL [31]. Probes were annotated using *meffil* [32]. Genome-wide significance was defined at the Bonferroni-corrected threshold of *p* < 1 × 10^−7^, and suggestive significance at *p* < 1 × 10^−5^. We included *p* < 1 × 10^−4^ specifically for pathway and enrichment analyses to allow a sufficient number of genes to be included.

We ran two sensitivity meta-analyses. First, we included only cohorts of predominantly European ancestry to check if the results of the main analysis were influenced by ancestry. Second, we performed leave-one-out meta-analyses for hits showing genome-wide significant associations with GPF to ensure that associations were not driven by a single cohort.

#### Differentially methylated regions

Differentially methylated regions (DMRs) were identified using the dmrff package [33] in R. This method first identifies candidate DMRs by screening the meta-level EWAS results for genomic regions each covered by a sequence of CpG sites with EWAS effects in the same direction, EWAS *p*-values<0.05, and <500 bp gaps between consecutive CpG sites. Then, summary statistics are calculated for each candidate DMR within each of the cohorts by meta-analyzing the cohort-level EWAS summary statistics of the CpG sites in the region. Meta-analysis is performed by a variation of inverse weighted fixed effects meta-analysis that accounts for non-independence between CpG sites. Finally, for each candidate DMR, the summary statistics from each cohort are meta-analyzed to obtain a crosscohort meta-analyzed DMR statistic and *p*-value.

#### Follow-up analyses

Individual probes showing genome-wide or suggestive significance were looked up in the EWAS catalog [34] and EWAS atlas [35] to examine potential associations with exposures and health outcomes based on existing studies. To further characterize potential environmental - as well as genetic – influences on these sites, we used two different tools: 1) a heritability tool quantifying additive genetic influences as opposed to shared and non-shared environmental influences on DNAm, based on data from monozygotic and dizygotic twins;[36] and 2) the GoDMC database (http://mqtldb.godmc.org.uk/) as a more specific tool for identifying genetic influences on DNAm levels via mQTL mapping. GoDMC is a large-scale collaborative effort including 36 cohorts (4 of which participated in this study: INMA, ALSPAC, GENR, GLAKU), based on whole blood from over 27,000 European samples. We characterized cross-tissue correspondence of DNAm using the Blood Brain DNA Methylation Comparison Tool by Hannon et al [37]., the Blood-Brain Epigenetic Concordance (BECon) [38], and the Iowa Methylation Array Graphing for Experimental Comparison of Peripheral tissue & Gray matter (IMAGE-CpG) [39]. To assess whether methylation levels of CpGs were associated with the expression levels of nearby genes in child blood, we consulted the HELIX Expression Quantitative Trait Methylation (eQTM) catalogue (https://helixomics.isglobal.org/), generated from samples overlapping with those included in this study (from the HELIX cohort). Finally, chromatin states associated to the most significant CpGs were assessed using ROADMAP blood 15 reference chromatin states (annotation and enrichment analysis conducted using the Enrichment module of the EASIER R package). Genome Browser (UCSC) was used to further explore the genomic context of the identified DMR.

To identify broader pathways and enrichment for molecular functions, we used the gene ontology (GO-biological processes, GO-molecular functions and GO-cellular components), the Kyoto Encyclopedia of Genes and Genomes (KEGG) pathway, and the Molecular Signature Database (MSigDB) enrichment methods from the missMethyl R package [40], as implemented in the Functional Enrichment module of the EASIER R package [41]. We ran GWAS enrichment analyses for EWAS using the GenomicRanges Package [42], to identify genomic regions of EWAS suggestive hits (*p* < 1 × 10^–4^) that overlapped with the 378 genome-wide significant loci previously reported in GWASs on general psychopathology [16], schizophrenia [43], neuroticism [44], ADHD [45] or anxiety [46] (0.5 Mb window centered to the genomic locus indicated in the original studies).

## Results

### General psychopathology factor

All mental health subscales had significant loadings on the general factor across all cohorts, with all loadings >0.30. For full details on the GPF loadings, correlations, and model fit, see Supplementary Table 1. The loadings of the mental health subscales on the specific internalizing and externalizing factors tended to be lower and were less consistent across the cohorts, as were the correlations between these specific factors. In INMA and HELIX, a model including the correlation between the specific internalizing and externalizing factors did not fit the data well (see Supplementary Information 1). Therefore, in both INMA and HELIX, the specific internalizing and externalizing factors were not allowed to correlate (i.e., completely orthogonal model; see Supplementary Table 1). In line with previous research [5, 7, 14], GPF consistently negatively correlated with child cognition (see Supplementary methods) across the cohorts (mean *r* = −0.12, range = −0.08 to −0.13).

### Epigenome-wide meta-analysis

**Fig. 1:**
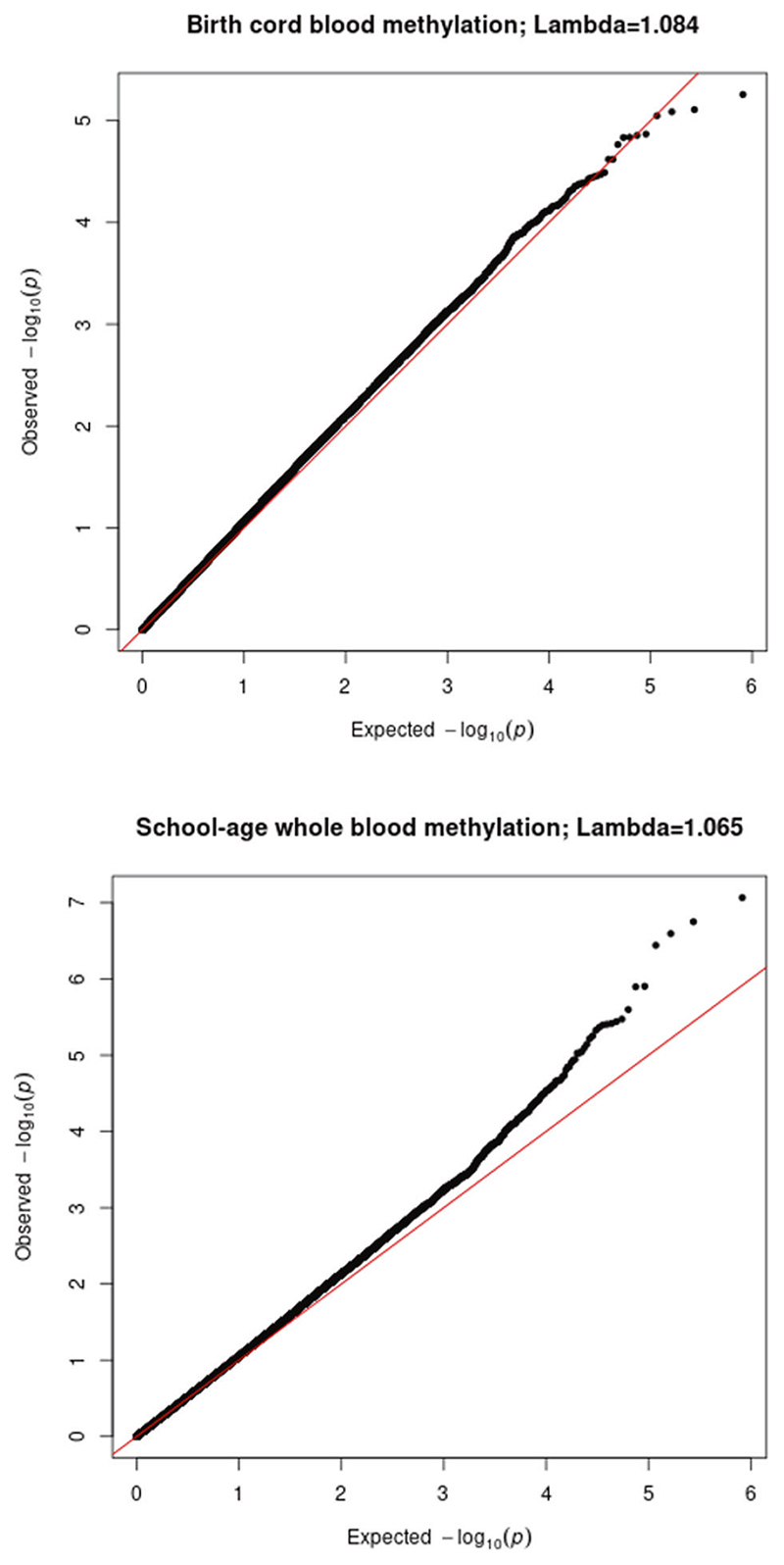
Quantile-quantile plot of the meta-analytic associations of DNA methylation at birth and DNA methylation at school-age with general psychopathology. The diagonal line represents the distribution of the expected p-values under the null. Points above the diagonal refer to p-values that are lower than expected.

**Fig. 2:**
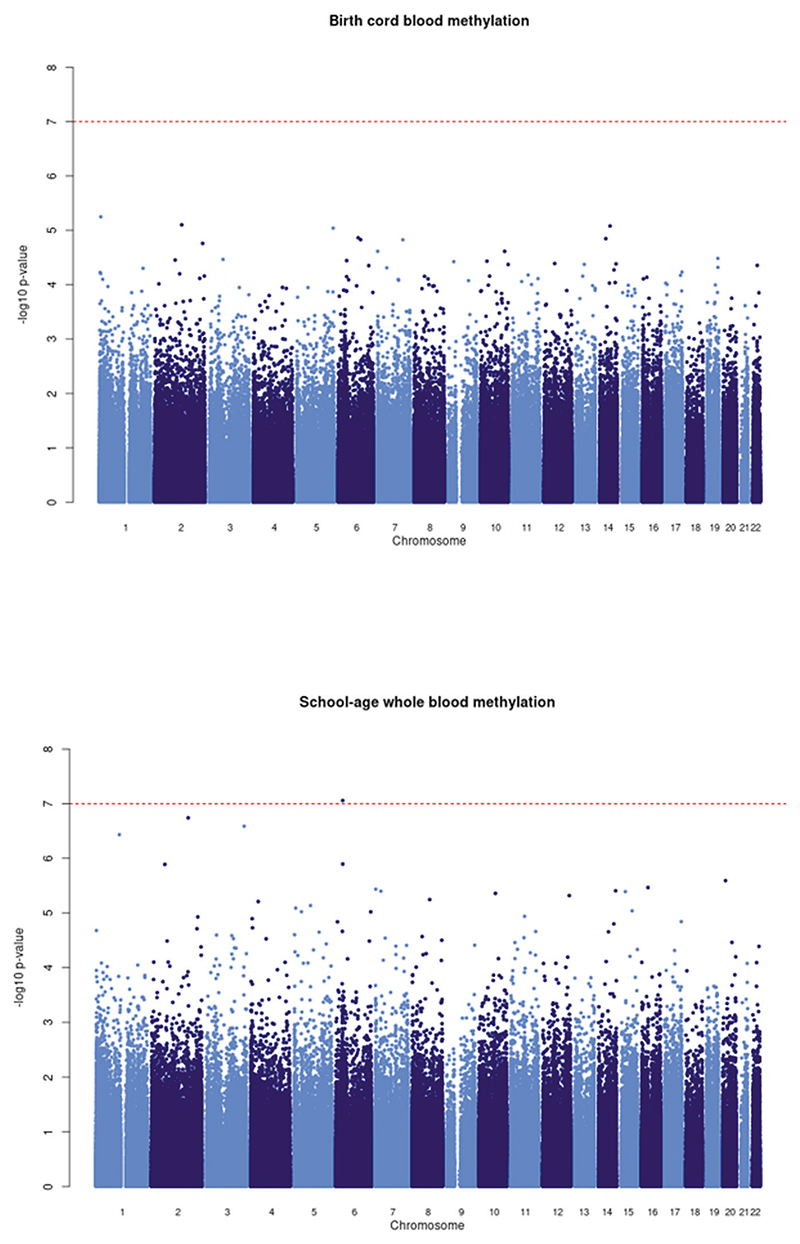
Manhattan plot of −log_10_ p-values versus CpG position (base pair and chromosome) showing meta-analytic associations of DNA methylation at birth and DNA methylation at school-age with general psychopathology. The red line indicates genome-wide significance (*p* < 1 × 10^−7^).

Descriptive statistics across the cohorts are shown in Supplementary Table 2. We note that some differences were observed in GPF levels and sociodemographic characteristics between the full cohort samples and analytical subsamples used in the present study (see Supplementary Table 2). These differences varied depending on the specific cohort and variable examined. We prospectively examined associations of DNAm at birth (*n* = 2178) at 404,017 CpG sites with GPF at school-age. There was no evidence of genomic inflation in the cohort-specific EWASs (range λ = 0.95–1.14), nor in the meta-analysis (λ = 1.08, see also Fig. 1). As can be seen in Fig. 2, no CpG reached genome-wide significance at *p* < 1 × 10^−7^, with four CpGs showing *p* < 1 × 10^−5^ (see Table 2). For the top hit (cg02084087), annotated to *TNFRSF25* (TNF Receptor Superfamily Member 25), a 10-point increase in percentage methylation was related to a 0.43 SD increase in general psychopathology symptoms (*p* = 5.54 × 10^−6^).

In the cross-sectional meta-analysis of DNAm at school-age (*n* = 2,190) at 413,497 sites, one CpG reached genome-wide significance (see Fig. 2). For this CpG probe (cg11945228), mapped to *BRD2* (Bromodomain-containing protein 2 gene), a 10-point increase in percentage methylation was related to a 3.70 SD decrease in general psychopathology symptoms (*p* = 8.58 × 10^−8^). Of note, there was a negative association between DNA methylation at this CpG and GPF in all cohorts except for the HELIX-Pakistani cohort. It is not possible based on the present data however to establish whether this may reflect an ancestry-specific association pattern or the influence of other cohort-specific factors (Supplementary Fig. 2). Twenty other CpGs showed *p* < 1 × 10^−5^ (see Table 3). These 21 top hits identified at school-age did not overlap with the ones observed at birth. Furthermore, as shown in Supplementary Table 3, the significant hit identified at school-age did not reach nominal significance (*p* < 0.05) at birth (B = 5.28, SE = 3.76, *p* = 0.16). Nominally significant probes identified in childhood correlated at *r* = 0.004, *p* = 0.55 (*n* = 23,764) with respective probes at birth.

#### Sensitivity analyses

Restricting the meta-analysis to children with European ancestry did not change the overall pattern of results for both prospective (*n*=2027) and cross-sectional (*n*=2125) studies, as evidenced by cross-meta-analysis correlations of effect estimates (*r*_prospective_ = 0.99, *r*_cross-sectional_ = 0.99) and consistent directions (95% and 96%, respectively) of effect estimates. The top hit identified at schoolage remained genome-wide significant (B=−38.02, SE=6.95, *p* = 4.47 × 10^−8^).

Leave-one-out meta-analyses showed that the significant top hit identified during childhood (cg11945228) was robust to excluding all individual cohorts, except GENR (for a leave-one-out plot, see Supplementary Fig. 1). Furthermore, when looking at the cohort-level EWAS results, the cross-sectional association between cg11945228 and GPF was statistically significant in GENR (B=−41.87, SE=7.78, *p* = 7.39 × 10^−8^) but not in the other cohorts (all *p* > 0.25, see Supplementary Fig. 2 for a forest plot).

Finally, we re-ran the cross-sectional EWAS analyses within HELIX using a different method to estimate cell-type proportions (i.e. based on Salas et al., 2018 [29] instead of Houseman et al., 2012 [28]. We found that the correlation between the regression beta coefficients for all CpGs was very high (r = 0.97) (Supplementary Fig. 3), indicating that results are highly concordant when using these two different methods.

### Differentially methylated regions

In the prospective analyses, there was no evidence of DMRs at birth associated with GPF. In the cross-sectional analyses, one DMR at childhood was associated with GPF (estimate = 10166.54, SE = 1800.19, *p* = 1.63 × 10^−8^). As shown in Supplementary Table 4, this DMR included 6 CpGs mapped to the gene body of the SHC Adaptor Protein 4 gene (*SHC4*) and close to the Transcription Start Site of the EP300 Interacting Inhibitor Of Differentiation 1 gene (*EID1*) at chromosome 15. From the 6 CpGs, 2 showed positive associations and 4 showed negative associations between methylation level and GPF.

### Follow-up analyses

All probes showing significant or suggestive associations with DNAm had twin heritability estimates available, showing mean additive genetic influences of *r*_birth_ = 0.16 and *r*_childhood_ = 9.44 × 10^−2^ (see Supplementary Table 5).

Of the four suggestive probes identified at birth, three were associated with at least one known methylation quantitative trait locus (mQTL) (see Supplementary Table 5a) and one (cg09437808) showed a concordant DNAm pattern (*r* > 0.28, *p* < 0.01) between blood and several brain regions (the prefrontal cortex, entorhinal cortex, and the superior temporal gyrus) according to Hannon et al. tool (see Supplementary Table 6a). This positive correlation between blood and brain is also reported by IMAGE CPG tool (*r* = 0.35, *p* = 0.31) (Supplementary Table 7a) but not identified in BECon.

The genome-wide significant probe identified during childhood (cg11945228) was unrelated to known mQTLs and showed non-significant correlations between blood and brain DNAm (data only available in one of the three online tools used to assess this concordance) (Supplementary Tables 5b and 6b). Of the 20 suggestive probes identified in childhood, ten were associated with at least one known mQTL (see Supplementary Table 5b) and four (cg22691524, cg09040034, cg25182716, cg18436008) showed a significant correlation between blood and at least one brain region DNAm (*r* > 0.25, *p* < 0.04; see Supplementary Table 6b) according to Hannon et al tool. These sites also showed positive correlations in the BECon (3 out of 4 CpGs; Supplementary Table 8b) and IMAGE CPG tool (3 of the 4 CpGs; Supplementary Table 7b). None of the suggestive probes identified at birth or childhood showed links to an eQTM. According to EWAS Atlas and EWAS Catalogue, methylation levels at these top CpGs seem to be variable and sensitive to age, sex, tissue, or substance exposure (smoking, alcohol, polychlorinated biphenyls), and/or associated to several traits such as inflammatory and neurological diseases (rheumatoid arthritis, Behcet’s disease, myalgic encephalomyelitis, multiple sclerosis, among others) (see Supplementary Table 9).

The six probes in the DMR within the childhood analyses showed low evidence of genetic effects, as indicated by both twin-based estimates (mean variance explained by additive genetic influences r = 0.007) and the lack of associations with known mQTLs. One probe, cg05867423, was positively correlated between blood and brain according to data from Hannon et al. tool (r = 0.36, *p* < 0.002), with positive correlations also identified in the BECon and IMAGE CPG tools (see Supplementary Tables 6c, 7c, 8c). In addition, cg08455700 showed high correlations (r = 0.71) between blood and Brodmann area 20 according to the BECon tool (Supplementary Table 7c). None of these probes was related to eQTMs in blood.

Regarding chromatin states, we found that the genome-wide significant probe (cg11945228) and the DMR found at childhood were associated with active states (active transcription start site (TSS)-proximal promoter state and a transcribed state at the 5’ and 3’ end of genes showing both promoter and enhancer signatures) (Supplementary Table 10). In fact, an enrichment analysis for chromatin states revealed an overrepresentation of active states associated with zinc finger protein genes (ZNF/Rpts) within the most significant CpGs (*p* < 1 × 10–4) detected in the prospective meta-analysis (Supplementary Table 11a). In contrast, no consistent enrichment for active states vs repressed states was found based on the most significant CpGs detected in the cross-sectional meta-analysis (*p* < 1 × 10–4). However, we observed a significant underrepresentation of active transcription start site (TSS)-proximal promoter states (TssAFlnk), and an overrepresentation of actively-transcribed states (Tx, TxWk) together with inactive quiescent states (Quies) (Supplementary Table 11b). Moreover, according to ENCODE data on several cell lines, including different blood cell types, the DMR (chr15:49,170,042-49,170,244, GRCh37/hg19) is enriched by H3K27Ac histone marks and overlaps with DNAse hypersensitive areas, which are usually associated to active regulatory elements (Supplementary Fig. 4a). Hence, according to Chromatin interaction data (in situ Hi-C Chromatin Structure from a lymphoblastoid cell line), the genomic elements comprised in the region involving the two genes associated with the DMR seem to strongly interact with each other (Supplementary Fig. 4b).

GO, KEGG, and MSigDB analyses revealed no significantly enriched common biological processes, cellular components, molecular functions or pathways for the genes mapped to the probes at *p* < 1 × 10^−4^ in the meta-analyses at birth (*n* = 56) and during childhood (*n* = 104) (see Supplementary Table 12a-f).

Results of the GWAS enrichment analyses for EWAS are presented in Supplementary Table 13. Of the 56 probes at *p* < 1 × 10^−4^ in the prospective EWAS meta-analysis, six overlapped with genomic loci previously linked to general psychopathology [16], schizophrenia [43], neuroticism [44], ADHD [45] or anxiety [46] based on GWASs. Of the 104 probes at *p* < 1 × 10^−4^ in our cross-sectional EWAS meta-analysis, 13 (12.5%) overlapped with genomic loci previously linked to these psychiatric outcomes. Most notably, this cross-sectional enrichment analysis prioritized cg08514304 (*TAOK2*), which was among the top 10 suggestive hits identified in our cross-sectional EWAS meta-analysis and showed a consistent direction of effect in all cohorts. Finally, regarding the DMR, SNPs within the region comprising the associated genes *SHC4* and *EID1* have been related with psychiatric disorders such as major depressive disorder [47, 48], bipolar disorder [49], mood and psychotic disorders [50], obsessive compulsive disorder [51], and schizophrenia [52] (Supplementary Table 14) (Supplementary Fig. 4b). Interestingly, both *SHC4* and *EID1* genes are highly expressed in the brain according to GTEx data (Supplementary Fig. 4).

## Discussion

We conducted the largest epigenome-wide meta-analysis of GPF in childhood, using DNAm assessments at two different time points (birth and childhood). The analyses revealed little evidence for probe-specific associations between DNAm in cord blood or peripheral blood and GPF. However, we did identify a significant DMR in childhood, implicating two relevant genes.

On the basis of probe-level genome-wide meta-analyses, we found that lower DNA methylation at cg11945228 at school-age was significantly associated with higher levels of GPF. Cg11945228 is located within the *BRD2* gene, a BET (bromodomains and extra terminal domain) family chromatin adaptor that controls the transcription of a wide range of pro-inflammatory genes [53] and is involved in neural tube closure [54], neurogenesis [55], and neuroinflammation [56]. DNAm of the *BRD2* promotor has been implicated in juvenile myoclonic epilepsy, a common adolescentonset genetic generalized epilepsy syndrome [57]. However, we advise caution when interpreting this specific site because, despite having low variation attributable to heterogeneity across the cohorts, its genome-wide significant association with GPF seems to be driven by one single cohort.

With regards to genes with probes at suggestive significance at school-age (*WDR20*, *MOV10*, and *TAOK2*), these have previously been linked to neurodevelopmental and psychiatric risk, such as autism spectrum disorder (ASD) and schizophrenia [58–65]. Pleiotropy was supported by our cross-sectional GWAS enrichment analyses for EWAS, showing that *TAOK2* overlapped with genomic loci previously linked to schizophrenia [16, 43], as well as obsessive compulsive disorder and bipolar disorder [16]. However, despite these previously established links with mental health outcomes, annotated genes of our overall top hits identified in the EWAS meta-analyses were not enriched for common biological processes, cellular components, molecular functions, or pathways.

The significant DMR identified at school-age included 6 CpGs mapped to *SHC4* and *EID1* genes, which are highly expressed in the brain. *SHC4* regulates BDNF-induced MAPK activation [66] and *EID1* plays an important role in the central nervous system [67], being involved in cell proliferation in the brain, synaptic plasticity and memory function. Interestingly, genetic variants in these genes have been previously implicated in multiple psychiatric disorders according to several studies (mostly GWAS), including bipolar disorder [49], obsessive-compulsive disorder [51], mood and psychotic disorders [50], schizophrenia [52], or MDD [47, 48, 68] (Supplementary Table 14) (Supplementary Fig. 4b). The fact that the DMR overlaps with active regulatory elements of these genes and shows evidence of blood-brain concordance for some of the CpGs supports the potential functional relevance of this region. Mechanistic studies will be needed in future to elucidate biological processes underlying the observed link between DNAm in this region and increased risk for multiple psychiatric outcomes. Of interest, despite similar sample sizes and measures (i.e., almost exclusively the CBCL), the top signals were very different between the prospective and cross-sectional EWASs, as evidenced for example by the lack of a correlation between nominally significant sites for these analyses. This low overlap might be due to the temporally dynamic nature of the methylome. DNAm patterns vary substantially over time [69] and can show temporally specific associations with outcomes, including psychiatric symptoms [20]. Unlike an existing EWAS meta-analysis on ADHD symptoms, which showed the strongest signal prospectively at birth compared to childhood [20], we did not detect any significant prospective associations. This is particularly interesting given the use of largely overlapping samples, suggesting that cord blood DNAm may capture risk for specific psychiatric problems (in this case ADHD) rather than a broader liability to psychopathology.

Strengths of this study include the large sample size and the use of DNAm at two different time points (birth and childhood), enabling the assessment of both prospective and cross-sectional associations with GPF. Another important strength is the use of standardized protocols and scripts to fit GPF to the data in a multicohort setting. The GPF scores we analyzed were previously found to associate with a module of co-methylated DNAm probes across the genome [23], suggesting that it is possible to detect biological correlates of GPF using this study’s measure. Furthermore, the current study showed that GPF consistently negatively correlated with child cognition across the cohorts as expected based on existing evidence [7], suggesting that it is capturing a similar, valid construct across the cohorts.

However, the current findings should also be interpreted in the context of several limitations. First, given the possibility of residual confounding and reverse causality, the direction of the observed associations cannot be inferred. DNAm might be a marker for unmeasured environmental factors that could affect GPF via independent pathways. Furthermore, children with higher levels of mental health problems may evoke a particular environment [70], which might affect DNAm. Second, our top hits were unrelated to eQTMs. Future research integrating transcriptomic data will be important for assessing the functional relevance of DNAm changes to gene expression in the brain. Third, because DNAm is tissue specific, our observations in peripheral blood may not reflect DNAm levels in other, potentially more relevant, tissues such as the brain. Despite potential sex differences in mental health problems [71] the current study did not examine sexspecificity for power reasons. Further, participating cohorts used different normalization pipelines, which may have contributed to cohort differences and influenced our results. In future, it would be optimal for meta-analytic studies to utilize a standardized processing pipeline across all samples. Furthermore, we found heterogeneity in CFA parameters, particularly for the specific internalizing and externalizing factors (especially in the GLAKU cohort). This precluded us from investigating whether, aside from the GPF, DNA methylation patterns also associate with variance that is unique to these symptom domains – an interesting question for future research. In future, it would be optimal for meta-analytic studies to utilize a standardized processing pipeline across all samples. Finally, the present findings are based on a predominantly European population and the cohorts are sampled from settings which largely have socialized healthcare, access to mental health services, and different cultural stigma surrounding mental health than other population groups. Future genome-wide studies with larger sample sizes are needed to replicate our findings in other ancestries and in more diverse settings to further characterize DNAm sites associated with GPF.

In summary, this large EWAS meta-analysis identified one probe (Cg11945228) for which lower DNAm in childhood was associated with higher levels of GPF. Furthermore, one DMR in childhood was associated with GPF. This DMR included 6 CpGs mapped to the *SHC4* gene that has previously been implicated in multiple types of psychiatric disorders in adulthood. In contrast, no prospective associations were identified with DNAm patterns at birth. The current findings call for a more integrative approach to the study of GPF, using multiple omics sources, including the genome, epigenome, and transcriptome, to achieve a more comprehensive understanding of its biological underpinnings.

## Supplementary Material

**Supplementary information** The online version contains supplementary material available at https://doi.org/10.1038/s41380-022-01871-6.

Supplementary Material

Supplementary Tables

## Figures and Tables

**Table 1:** Cohort characteristics.

Cohort	Ancestry/ethnicity	*n*	DNAm age	GPF age	GPF instrument	Effect sizes			λ
						1st Qu	Median	3rd Qu	
*Birth EWAS*									
ALSPAC	European	643	0	10	DAWBA	−1.10	0.02	1.18	0.98
DCHS	Black African, mixed	151	0	6	CBCL	−4.35	−0.77	2.75	0.95
Generation R	European	1092	0	10	CBCL	−0.77	0.08	1.00	1.01
INMA	European	292	0	9	CBCL	−1.53	0.39	3.03	1.14
Meta		2178				−0.51	0.07	0.77	1.08
*Childhood EWAS*									
ALSPAC	European	697	7	10	DAWBA	−1.26	0.02	1.28	1.00
Generation R	European	434	10	10	CBCL	−1.51	0.08	1.84	1.03
GLAKU	European	215	12	12	CBCL	−1.66	0.31	2.24	0.91
HELIX	European	779	8	8	CBCL	−1.36	0.32	2.22	0.99
HELIX	Pakistani	65	7	7	CBCL	−4.47	2.52	10.95	1.20
Meta		2190				−0.54	0.18	1.00	1.07

**Table 2:** DNA methylation at birth and general psychopathology: meta-analytic associations with *p* < 1 × 10^−5^

CpG	Gene	Chr	Position	n	B	SE	p	Direction	I^2^	Heterogeneity *p*-value
cg02084087	*TNFRSF25*	chr1	6526049	2175	4.31	0.95	5.54 × 10^−6^	++++	46.2	0.13
cg11777523	*GPR148*	chr2	131485418	2019	9.26	2.07	7.80 × 10^−6^	+?++	23.6	0.27
cg14358879	*SLC8A3*	chr14	70655920	2174	-7.82	1.75	8.20 × 10^−6^	----	33.7	0.21
cg09437808	-	chr5	176107069	2177	2.46	0.55	8.96 × 10^−6^	++++	0	0.47

*Chr* chromosome, *n* number of participants, *SE* standard error, *Direction* direction of the effect per study (ALSPAC, DCHS, GENR, INMA) in alphabetical order (+ = positive direction, − = negative direction, ? = not present); *I^2^* heterogeneity statistic reflecting the variation attributable to heterogeneity across studies (high values suggest high heterogeneity)

Effect estimates (B) represent the SD increase in GPF for each increase of 100% in DNAm.

**Table 3:** DNA methylation at school-age and general psychopathology: meta-analytic associations with *p* < 1 × 10^−5^

CpG	Gene	Chr	Position	n	B	SE	p	Direction	I^2^	Heterogeneity *p*- value
cg11945228	*BRD2*	chr6	32940368	2173	-37.00	6.91	8.58 × 10^−8^	----+	0	0.53
cg18862005	-	chr2	177940863	1972	3.78	0.72	1.78 × 10^−7^	++?++	0	0.64
cg22691524	-	chr3	185300576	2180	7.84	1.52	2.54 × 10^−7^	+++++	0	0.47
cg00719568	*MOV10*	chr1	113239645	2184	4.20	0.83	3.62×10^−7^	+++++	0	0.67
cg09040034	*KIFC1*	chr6	33362567	1966	4.89	1.01	1.25 × 10^−6^	++?+−	40	0.17
cg24514921	*VPS54*	chr2	64246311	2184	12.17	2.51	1.27 × 10^−6^	++++−	0	0.45
cg25182716	-	chr20	13622875	2178	6.42	1.36	2.52 × 10^−6^	+++++	0	0.76
cg08514304	*TAOK2*	chr16	29994437	2175	6.88	1.48	3.36 × 10^−6^	+++++	0	0.59
cg00470277	-	chr7	2669915	2185	4.55	0.98	3.62 × 10^−6^	+++++	0	0.99
cg26353764	*WDR20*	chr14	102660055	2187	6.82	1.48	3.86 × 10^−6^	+++++	43.7	0.13
cg27009703	*HOXA9*	chr7	27204894	2121	-8.58	1.86	3.93 × 10^−6^	-----	0	0.52
cg12492087	*ZFP106*	chr15	42749885	2178	6.39	1.39	4.01 × 10^−6^	+++++	0	0.92
cg18436008	-	chr10	80535327	2176	5.48	1.19	4.31 × 10^−6^	+++++	0	0.56
cg17281031	-	chr12	128223216	2186	4.50	0.98	4.73 × 10^−6^	+++++	14.6	0.32
cg08327106	*RALYL*	chr8	85094842	2175	8.60	1.89	5.60 × 10^−6^	+++++	0	0.66
cg11236841	-	chr4	35567978	2175	4.67	1.03	6.08 × 10^−6^	+++++	54.3	0.07
cg21525176	*LHFPL2*	chr5	77906752	2182	6.09	1.36	7.19 × 10^−6^	++−++	59.2	0.04
cg26420013	*NSUN2;SRD5A1*	chr5	6632020	2189	2.44	0.55	8.03 × 10^−6^	+++++	56.1	0.06
cg17087232	*MAN2C1*	chr15	75651821	2187	3.45	0.78	9.00 × 10^−6^	+++++	0	0.98
cg16360861	*RAI14*	chr5	34684597	2175	5.12	1.16	9.36 × 10^−6^	+++++	0	0.53
cg00737264	*SMOC2*	chr6	169049498	2188	1.88	0.42	9.40 × 10^−6^	+−+++	41.3	0.15

*Chr* chromosome, *n* number of participants, *SE* standard error, *Direction* direction of the effect per study (ALSPAC, GENR, GLAKU, HELIX, HELIX-Pakistani) in alphabetical order (+= positive direction, −= negative direction, ? = not present); *I*^2^ heterogeneity statistic reflecting the variation attributable to heterogeneity across studies (high values suggest high heterogeneity)

Effect estimates (B) represent the SD increase in GPF for each increase of 100% in DNAm.

## Data Availability

Site-level meta-analytical results will be made publicly available (Supplementary data file) upon acceptance for publication. For access to cohort-level data, requests can be sent directly to individual studies.

## Code Availability

Analytical codes can be requested from authors.

## References

[1] Angold A, Costello EJ, Erkanli A (1999). Comorbidity. J Child Psychol Psychiatry.

[2] Kessler RC, Chiu WT, Demler O, Merikangas KR, Walters EE (2005). Prevalence, severity, and comorbidity of 12-month DSM-IV disorders in the National Comorbidity Survey Replication. Arch Gen Psychiatry.

[3] Cuffe SP, Visser SN, Holbrook JR, Danielson ML, Geryk LL, Wolraich ML (2020). ADHD and Psychiatric Comorbidity: Functional Outcomes in a School-Based Sample of Children. J Atten Disord.

[4] Roy-Byrne PP, Stang P, Wittchen HU, Ustun B, Walters EE, Kessler RC (2000). Lifetime panic-depression comorbidity in the National Comorbidity Survey. Association with symptoms, impairment, course and help-seeking. Br J Psychiatry J Ment Sci.

[5] Lahey BB, Rathouz PJ, Keenan K, Stepp SD, Loeber R, Hipwell AE (2015). Criterion validity of the general factor of psychopathology in a prospective study of girls. J Child Psychol Psychiatry.

[6] Rijlaarsdam J, Cecil CAM, Buil JM, van Lier PAC, Barker ED (2021). Exposure to Bullying and General Psychopathology: A Prospective, Longitudinal Study. Res Child Adolesc Psychopathol.

[7] Neumann A, Pappa I, Lahey BB, Verhulst FC, Medina-Gomez C, Jaddoe VW (2016). Single Nucleotide Polymorphism Heritability of a General Psychopathology Factor in Children. J Am Acad Child Adolesc Psychiatry.

[8] Caspi A, Moffitt TE (2018). All for One and One for All: Mental Disorders in One Dimension. Am J Psychiatry.

[9] Pettersson E, Lahey BB, Larsson H, Lichtenstein P (2018). Criterion Validity and Utility of the General Factor of Psychopathology in Childhood: Predictive Associations With Independently Measured Severe Adverse Mental Health Outcomes in Adolescence. J Am Acad Child Adolesc Psychiatry.

[10] Sallis H, Szekely E, Neumann A, Jolicoeur-Martineau A, van IJzendoorn M, Hillegers M (2019). General psychopathology, internalising and externalising in children and functional outcomes in late adolescence. J Child Psychol Psychiatry.

[11] Brikell I, Larsson H, Lu Y, Pettersson E, Chen Q, Kuja-Halkola R (2020). The contribution of common genetic risk variants for ADHD to a general factor of childhood psychopathology. Mol Psychiatry.

[12] Riglin L, Thapar AK, Leppert B, Martin J, Richards A, Anney R (2020). Using Genetics to Examine a General Liability to Childhood Psychopathology. Behav Genet.

[13] Brodbeck J, Fassbinder E, Schweiger U, Fehr A, Späth C, Klein JP (2018). Differential associations between patterns of child maltreatment and comorbidity in adult depressed patients. J Affect Disord.

[14] Caspi A, Houts RM, Belsky DW, Goldman-Mellor SJ, Harrington H, Israel S (2014). The p Factor: One General Psychopathology Factor in the Structure of Psychiatric Disorders? Clin Psychol Sci J Assoc. Psychol Sci.

[15] Campbell M, Jahanshad N, Mufford M, Choi KW, Lee P, Ramesar R (2021). Overlap in genetic risk for cross-disorder vulnerability to mental disorders and genetic risk for altered subcortical brain volumes. J Affect Disord.

[16] Cross-Disorder Group of the Psychiatric Genomics Consortium. plee0@mgh.harvard.edu, Cross-Disorder Group of the Psychiatric Genomics Consortium (2019). Genomic Relationships, Novel Loci, and Pleiotropic Mechanisms across Eight Psychiatric Disorders. Cell.

[17] Teschendorff AE, Relton CL (2018). Statistical and integrative system-level analysis of DNA methylation data. Nat Rev Genet.

[18] Meaney MJ (2010). Epigenetics and the biological definition of gene x environment interactions. Child Dev.

[19] Cecil CAM, Walton E, Jaffee SR, O’Connor T, Maughan B, Relton CL (2018). Neonatal DNA methylation and early-onset conduct problems: A genome-wide, prospective study. Dev Psychopathol.

[20] Neumann A, Walton E, Alemany S, Cecil C, González JR, Jima DD (2020). Association between DNA methylation and ADHD symptoms from birth to school age: a prospective meta-analysis. Transl Psychiatry.

[21] Hannon E, Dempster E, Viana J, Burrage J, Smith AR, Macdonald R (2016). An integrated genetic-epigenetic analysis of schizophrenia: evidence for colocalization of genetic associations and differential DNA methylation. Genome Biol.

[22] Zhu Y, Strachan E, Fowler E, Bacus T, Roy-Byrne P, Zhao J (2019). Genome-wide profiling of DNA methylome and transcriptome in peripheral blood monocytes for major depression: A Monozygotic Discordant Twin Study. Transl Psychiatry.

[23] Rijlaarsdam J, Barker ED, Caserini C, Koopman-Verhoeff ME, Mulder RH, Felix JF (2021). Genome-wide DNA methylation patterns associated with general psychopathology in children. J Psychiatr Res.

[24] Zhou W, Laird PW, Shen H (2017). Comprehensive characterization, annotation and innovative use of Infinium DNA methylation BeadChip probes. Nucl Acids Res.

[25] Solomon O, MacIsaac J, Quach H, Tindula G, Kobor MS, Huen K (2018). Comparison of DNA methylation measured by Illumina 450K and EPIC BeadChips in blood of newborns and 14-year-old children. Epigenetics.

[26] Patalay P, Fonagy P, Deighton J, Belsky J, Vostanis P, Wolpert M (2015). A general psychopathology factor in early adolescence. Br J Psychiatry J Ment Sci.

[27] Gervin K, Salas LA, Bakulski KM, van Zelm MC, Koestler DC, Wiencke JK (2019). Systematic evaluation and validation of reference and library selection methods for deconvolution of cord blood DNA methylation data. Clin Epigenetics.

[28] Houseman EA, Accomando WP, Koestler DC, Christensen BC, Marsit CJ, Nelson HH (2012). DNA methylation arrays as surrogate measures of cell mixture distribution. BMC Bioinforma.

[29] Salas LA, Koestler DC, Butler RA, Hansen HM, Wiencke JK, Kelsey KT (2018). An optimized library for reference-based deconvolution of whole-blood biospecimens assayed using the Illumina HumanMethylationEPIC BeadArray. Genome Biol.

[30] Rosseel Y (2012). lavaan: An R Package for Structural Equation Modeling. J Stat Softw.

[31] Willer CJ, Li Y, Abecasis GR (2010). METAL: fast and efficient meta-analysis of genome-wide association scans. Bioinforma Oxf Engl.

[32] Min JL, Hemani G, Davey Smith G, Relton C, Suderman M (2018). Meffil: efficient normalization and analysis of very large DNA methylation datasets. Bioinforma Oxf Engl.

[33] Suderman M, Staley JR, French R, Arathimos R, Simpkin A, Tilling K (2018). dmrff: identifying differentially methylated regions efficiently with power and control. bioRxiv.

[34] Battram T, Yousefi P, Crawford G, Prince C, Sheikhali Babaei M, Sharp G (2022). The EWAS Catalog: a database of epigenome-wide association studies. Wellcome Open Res.

[35] Li M, Zou D, Li Z, Gao R, Sang J, Zhang Y (2019). EWAS Atlas: a curated knowledgebase of epigenome-wide association studies. Nucleic Acids Res.

[36] Hannon E, Knox O, Sugden K, Burrage J, Wong CCY, Belsky DW (2018). Characterizing genetic and environmental influences on variable DNA methylation using monozygotic and dizygotic twins. PLoS Genet.

[37] Hannon E, Lunnon K, Schalkwyk L, Mill J (2015). Interindividual methylomic variation across blood, cortex, and cerebellum: implications for epigenetic studies of neurological and neuropsychiatric phenotypes. Epigenetics.

[38] Edgar RD, Jones MJ, Meaney MJ, Turecki G, Kobor MS (2017). BECon: a tool for interpreting DNA methylation findings from blood in the context of brain. Transl Psychiatry.

[39] Braun P, Han S, Nagahama Y, Gaul L, Heinzman J, Hing B (2019). 28 - IMAGE-CpG: DEVELOPMENT OF A WEB-BASED SEARCH TOOL FOR GENOME-WIDE DNA METHYLATION CORRELATION BETWEEN LIVE HUMAN BRAIN AND PERIPHERAL TISSUES WITHIN INDIVIDUALS. Eur Neuropsychopharmacol.

[40] Phipson B, Maksimovic J, Oshlack A (2016). missMethyl: an R package for analyzing data from Illumina’s HumanMethylation450 platform. Bioinforma Oxf Engl.

[41] isglobal-brge/EASIER: EwAS: quality control, meta-analysIs and EnRichment version 0128 from GitHub.

[42] Lawrence M, Huber W, Pagès H, Aboyoun P, Carlson M, Gentleman R (2013). Software for computing and annotating genomic ranges. PLoS Comput Biol.

[43] Schizophrenia Working Group of the Psychiatric Genomics Consortium (2014). Biological insights from 108 schizophrenia-associated genetic loci. Nature.

[44] Nagel M, Jansen PR, Stringer S, Watanabe K, de Leeuw CA, Bryois J (2018). Metaanalysis of genome-wide association studies for neuroticism in 449,484 individuals identifies novel genetic loci and pathways. Nat Genet.

[45] Demontis D, Walters RK, Martin J, Mattheisen M, Als TD, Agerbo E (2019). Discovery of the first genome-wide significant risk loci for attention deficit/hyperactivity disorder. Nat Genet.

[46] Levey DF, Gelernter J, Polimanti R, Zhou H, Cheng Z, Aslan M (2020). Reproducible Genetic Risk Loci for Anxiety: Results From ~200,000 Participants in the Million Veteran Program. Am J Psychiatry.

[47] Aragam N, Wang K-S, Pan Y (2011). Genome-wide association analysis of gender differences in major depressive disorder in the Netherlands NESDA and NTR population-based samples. J Affect Disord.

[48] Howard DM, Hall LS, Hafferty JD, Zeng Y, Adams MJ, Clarke T-K (2017). Genomewide haplotype-based association analysis of major depressive disorder in Generation Scotland and UK Biobank. Transl Psychiatry.

[49] Budde M, Friedrichs S, Alliey-Rodriguez N, Ament S, Badner JA, Berrettini WH (2019). Efficient region-based test strategy uncovers genetic risk factors for functional outcome in bipolar disorder. Eur Neuropsychopharmacol J Eur Coll Neuropsychopharmacol.

[50] Blokland GAM, Grove J, Chen C-Y, Cotsapas C, Tobet S, Handa R (2022). Sex-Dependent Shared and Nonshared Genetic Architecture Across Mood and Psychotic Disorders. Biol Psychiatry.

[51] Boraska V, Davis OSP, Cherkas LF, Helder SG, Harris J, Krug I (2012). Genome-wide association analysis of eating disorder-related symptoms, behaviors, and personality traits. Am J Med Genet Part B Neuropsychiatr Genet Publ Int Soc Psychiatr Genet.

[52] Goes FS, McGrath J, Avramopoulos D, Wolyniec P, Pirooznia M, Ruczinski I (2015). Genome-wide association study of schizophrenia in Ashkenazi Jews. Am J Med Genet Part B Neuropsychiatr Genet Publ Int Soc Psychiatr Genet.

[53] Wang N, Wu R, Tang D, Kang R (2021). The BET family in immunity and disease. Signal Transduct Target Ther.

[54] Gyuris A, Donovan DJ, Seymour KA, Lovasco LA, Smilowitz NR, Halperin ALP (2009). The chromatin-targeting protein Brd2 is required for neural tube closure and embryogenesis. Biochim Biophys Acta.

[55] Garcia-Gutierrez P, Juarez-Vicente F, Wolgemuth DJ, Garcia-Dominguez M (2014). Pleiotrophin antagonizes Brd2 during neuronal differentiation. J Cell Sci.

[56] DeMars KM, Yang C, Candelario-Jalil E (2019). Neuroprotective effects of targeting BET proteins for degradation with dBET1 in aged mice subjected to ischemic stroke. Neurochem Int.

[57] Pathak S, Miller J, Morris EC, Stewart WCL, Greenberg DA (2018). DNA methylation of the BRD2 promoter is associated with juvenile myoclonic epilepsy in Caucasians. Epilepsia.

[58] Wockner LF, Noble EP, Lawford BR, Young RM, Morris CP, Whitehall VLJ (2014). Genome-wide DNA methylation analysis of human brain tissue from schizophrenia patients. Transl Psychiatry.

[59] McKinney B, Ding Y, Lewis DA, Sweet RA (2017). DNA methylation as a putative mechanism for reduced dendritic spine density in the superior temporal gyrus of subjects with schizophrenia. Transl Psychiatry.

[60] Skariah G, Seimetz J, Norsworthy M, Lannom MC, Kenny PJ, Elrakhawy M (2017). Mov10 suppresses retroelements and regulates neuronal development and function in the developing brain. BMC Biol.

[61] Vissers LELM, Gilissen C, Veltman JA (2016). Genetic studies in intellectual disability and related disorders. Nat Rev Genet.

[62] Hanson E, Bernier R, Porche K, Jackson FI, Goin-Kochel RP, Snyder LG (2015). The cognitive and behavioral phenotype of the 16p11.2 deletion in a clinically ascertained population. Biol Psychiatry.

[63] Zufferey F, Sherr EH, Beckmann ND, Hanson E, Maillard AM, Hippolyte L (2012). A 600 kb deletion syndrome at 16p11.2 leads to energy imbalance and neuropsychiatric disorders. J Med Genet.

[64] Weiss LA, Shen Y, Korn JM, Arking DE, Miller DT, Fossdal R (2008). Association between microdeletion and microduplication at 16p11.2 and autism. N Engl J Med.

[65] Chang H, Li L, Li M, Xiao X (2017). Rare and common variants at 16p11.2 are associated with schizophrenia. Schizophr Res.

[66] You Y, Li W, Gong Y, Yin B, Qiang B, Yuan J (2010). ShcD interacts with TrkB via its PTB and SH2 domains and regulates BDNF-induced MAPK activation. BMB Rep.

[67] Liu R, Lei JX, Luo C, Lan X, Chi L, Deng P (2012). Increased EID1 nuclear translocation impairs synaptic plasticity and memory function associated with pathogenesis of Alzheimer’s disease. Neurobiol Dis.

[68] Sullivan PF, de Geus EJC, Willemsen G, James MR, Smit JH, Zandbelt T (2009). Genome-wide association for major depressive disorder: a possible role for the presynaptic protein piccolo. Mol Psychiatry.

[69] Mulder RH, Neumann A, Cecil CAM, Walton E, Houtepen LC, Simpkin AJ (2021). Epigenome-wide change and variation in DNA methylation in childhood: trajectories from birth to late adolescence. Hum Mol Genet.

[70] Singham T, Viding E, Schoeler T, Arseneault L, Ronald A, Cecil CM (2017). Concurrent and Longitudinal Contribution of Exposure to Bullying in Childhood to Mental Health: The Role of Vulnerability and Resilience. JAMA Psychiatry.

[71] Bale TL, Epperson CN (2017). Sex as a Biological Variable: Who, What, When, Why, and How. Neuropsychopharmacol Publ Am Coll Neuropsychopharmacol.

